# The Emerging Role of H3K9me3 as a Potential Therapeutic Target in Acute Myeloid Leukemia

**DOI:** 10.3389/fonc.2019.00705

**Published:** 2019-08-02

**Authors:** Laura Monaghan, Matthew E. Massett, Roderick P. Bunschoten, Alex Hoose, Petrisor-Alin Pirvan, Robert M. J. Liskamp, Heather G. Jørgensen, Xu Huang

**Affiliations:** ^1^Haemato-Oncology/Systems Medicine Group, Paul O'Gorman Leukemia Research Center, Institute of Cancer Sciences, University of Glasgow, Glasgow, United Kingdom; ^2^School of Chemistry, University of Glasgow, Glasgow, United Kingdom

**Keywords:** acute myeloid leukemia (AML), Leukaemic stem cell (LSC), Histone 3 lysine 9 trimethylation (H3K9me3), lysine specific demethylase (KDM), heterochromatin, gene suppression

## Abstract

Growing evidence has demonstrated that epigenetic dysregulation is a common pathological feature in human cancer cells. Global alterations in the epigenetic landscape are prevalent in malignant cells across different solid tumors including, prostate cancer, non-small-cell lung cancer, renal cell carcinoma, and in haemopoietic malignancy. In particular, DNA hypomethylation and histone hypoacetylation have been observed in acute myeloid leukemia (AML) patient blasts, with histone methylation being an emerging area of study. Histone 3 lysine 9 trimethylation (H3K9me3) is a post-translational modification known to be involved in the regulation of a broad range of biological processes, including the formation of transcriptionally silent heterochromatin. Following the observation of its aberrant methylation status in hematological malignancy and several other cancer phenotypes, recent studies have associated H3K9me3 levels with patient outcome and highlighted key molecular mechanisms linking H3K9me3 profile with AML etiology in a number of large-scale meta-analysis. Consequently, the development and application of small molecule inhibitors which target the histone methyltransferases or demethylase enzymes known to participate in the oncogenic regulation of H3K9me3 in AML represents an advancing area of ongoing study. Here, we provide a comprehensive review on how this particular epigenetic mark is regulated within cells and its emerging role as a potential therapeutic target in AML, along with an update on the current research into advancing the generation of more potent and selective inhibitors against known H3K9 methyltransferases and demethylases.

## Background

The normal process of haematopoietic progression involves the differentiation and self-renewal of haematopoietic stem cells (HSC), as a hierarchical tree the development and progression of blood cells has become a model organism for the study of how the epigenetic landscape changes with cell commitment and ultimately its involvement in cell fate decision making. The precisely controlled expression of lineage specific genes is crucial for the proliferation and differentiation essential for normal development, whereas aberrant transcriptional regulation can result in malignant transformation and oncogenic progression.

The regulation of gene transcription is a tightly controlled process in which DNA associated with a number of transcription factors and histone proteins forms the structure of chromatin, each component playing a role in regulating the gene expression landscape. Epigenetics concerns modification to the marks associates with the DNA and histones which are not mutations but rather alter the structure of the chromatin making genes more or less accessible whilst also recruiting proteins which serve to alter the chromatin structure and promote transcription (1). In 1962, two decades after the coining of the epigenome by Conrad Waddington in 1942 (2), histone methylation was first postulated to be involved with gene expression (3). Over 50 years on and considerable progress has been made in understanding the role of methylated histones. A collection of studies within the first decade of the twenty-first century presented a turning point for our understanding of histone methylation, transforming it from a static histone modification to a dynamic mark whose abundance could be externally altered. Whilst the first methyltransferase SUV39H1 was identified at the turn of the century (4) and hinted that histone methylation was dynamic, it was arguably the subsequent discovery of the first histone demethylase (5) that shifted our perspective of histone methylation. This discovery revealed that the marks were not permanent and paved the way for future studies concerning the regulation of specific methylated histones and their impact on normal tissue development and oncogenic transformation.

Acute Myeloid Leukemia (AML) is an aggressive hematological malignancy characterized by disruption of the normal process of haematopoietic progression involving the differentiation and self-renewal of HSC. These lead to the uncontrolled proliferation of dominant Leukaemic stem cell (LSC) clones and the accumulation of immature myeloid blasts. Originally AML was thought to progress through the two-hit hypothesis in which the acquisition of a type 1 mutation results in an increased rate of proliferation and resistance to apoptosis in collaboration with a type 2 mutation which blocks differentiation (6). With increasing understanding of the epigenome, a third subgroup has been added featuring aberrant epigenetic regulation (7). Genome-wide and candidate gene association studies have highlighted mutations associated with over 70% of *de novo* AML occurring in genes encoding for epigenetic regulators (8–12), further highlighting the crucial role that epigenetic modifications plays in hematological malignancy.

Moreover, in primary cell sample analysis on comparison of AML samples with normal CD34^+^ stem and progenitor cells and white blood cells as controls, a preferential decrease in H3K9me3 at core promotor regions is observed in AML blasts, this fundamentally has causal transcription factor changes recruiting varying histone modifying enzymes and altering the chromatin structure (13). Over 2000 loci differed between the sample groups with ~20% of these with a higher than 2-fold change. Fundamentally the changes observed could be significantly centered on the region from −300 to the transcriptional start site and clearly allowed the separation of distinct sample groups (13). By investigating how these enzymes become deregulated in AML and identifying ways to target these, new avenues begin to emerge for possible treatment options to complement the mainstay chemotherapy strategy. In this review, we draw our attention to one particular epigenetic mark, the trimethylation of lysine 9 residues on histone 3 (H3K9me3) which is highly regulated in the normal HSC differentiation and self-renewal alongside the establishment of pre-LSCs (14). A wide range of enzymes are involved in its establishment, recognition, and removal, representing promising therapeutic targets in AML.

## Main Text

### Role of H3K9me3 in Normal Haematopoiesis and Malignancy

H3K9me3 has a role not only in malignancy but in normal cellular development, acting as a repressor of lineage inappropriate genes and maintaining early cell integrity and genomic stability. In the early 2000's a number of groups provided evidence of its importance in interacting with the evolutionarily conserved amino terminal chromodomain of heterochromatin protein 1 (HP1), a hallmark of heterochromatin, thereby recruiting it to specific chromatin loci (15–17). Heterochromatin is a unique form of chromatin architecture defined as condensed and transcriptionally silent (18). Crucially, HP1 can cause deposition of further H3K9me3 through the recruitment of the methyltransferase SUV39H1 (19) leading to propagation of H3K9me3 across DNA and permitting the establishment of large domains of heterochromatin (20). Fundamental for cell integrity and maintenance, large constitutive heterochromatin facilitated by H3K9me3 maintains repetitive gene clusters and regulatory factors and prevents the unwanted recombination and introduction of mutations. In mouse models, reducing or knocking out the fundamental H3 methyltransferases cause lethality to the embryos at different stages of development (21). By binding with HP1 protein, H3K9me3 recruits further epigenetic modifications which contribute to the maintenance of the heterochromatin (22). However, in facilitative heterochromatin accessing the genes is fundamental for the changing cellular landscape when the cell is forced to adapt, for this reason the mark and subsequent tightly bound heterochromatin must be closely regulated (23). This has been explored through the use of several models which detail the reactions kinetics (24, 25). To date, roles for H3K9me3 have been discovered in regulating apoptosis (26, 27), autophagy (28), development (29, 30), DNA repair (31–35), splicing (36–38), self-renewal (39, 40), transcriptional elongation (41), viral latency (42–44), imprinting (45), aging (46), and cell identity (47).

The role of H3K9me3 within cells is still being explored and has proved to be multi-faceted and intricate in a wide-range of cellular processes, which consequently highlight an extended network of mechanisms which contribute to its accumulation and subsequent genomic stability. H3K9me3 has been implicated in recent years with the progression of pancreatic metastasis, with the somatic mutation burden remaining primarily unchanged within the metastasis a global change is observed in H3K9 methylation status giving a selective advantage and an increased capacity to survive and resist treatment to the metastatic site (48). In AML alterations of H3K9 methylation at promoter regions are associated with an inactivation of tumor suppressor genes and a blockage in differentiation and deregulated proliferation (49, 50). Given the reversible nature of H3K9 trimethylation, this represents an attractive therapeutic target in AML.

### The Association of H3K9me3 With Transcriptional Activation

The modifications of histones must not be thought of alone but as a small part of a bigger picture of regulation. Chromatin is not an open and closed book as once was thought but is subject to a number of regulatory factors and enzymes which together influence the outlook. Histone modifications can influence the chromatin structure without direct chromatin access changes (51).An alternative approach to investigating the histone code has identified an opposing role for H3K9me3 in AML. Some evidence postulates a role for H3K9me3 as associated with activation either as a solo mark by selectively interacting with RNA polymerase II to promote mRNA elongation and transcriptional activation or through a specific histone code co-localizing on fundamental genes with activating histone marks H3K9ac and H3K4me2 (52, 53). Similarly, H3K9me3's binding capacity for HP1 can also be questioned as to its mechanism and consequence. HP1 protein exists in three forms α,β, and γ. By binding the α and β subtypes the classical heterochromatin transcriptional repression phenotype is observed but when the mark can be co-localized with the third form HP1γ with 1 kb of the transcriptional start site (TSS) there is RNA polymerase II induced mRNA elongation promoting gene activation which is rapidly lost upon transcriptional termination. This is most clearly seen during the constitutive expression of GAPDH observed with a firm H3K9me3 HP1γ binding phenotype at its TSS (41).

### Regulatory Mechanisms of H3K9me3

**Figure 1:**
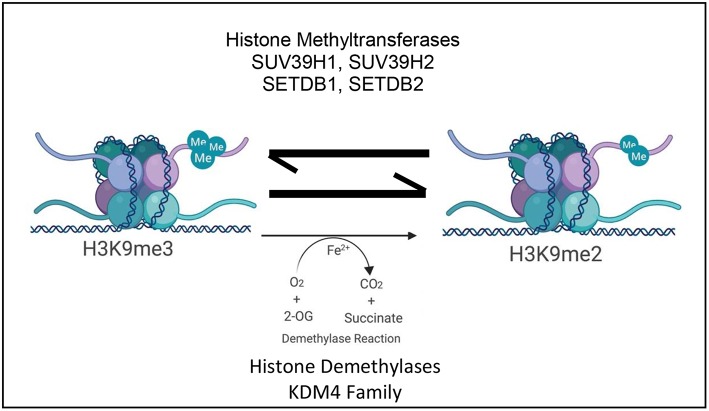
Schematic depicting the current paradigm of H3K9me3 regulation and its influence on transcription by the antagonistic activity of various histone lysine methyltransferases and the KDM4 family of histone lysine specific demethylases ©Created with Biorender.com.

The dynamic nature of H3K9me3 highlights the number of enzymes which are involved in its regulation. A tightly controlled collection of readers, writers, and erasers of this histone mark establish and maintain transcriptional landscape however small changes in these enzymes can also consequently contribute to disease (Figure 1; Table 1).

**Table 1:** Representative summary of enzymes involved in the regulation of H3K9 methylaton—their target state, function, and role in the development of disease.

**Enzyme**	**Role**	**Target**	
SUV39H1	Methyltransferase	H3K9me3	Relocation of genes into heterochromatin and transcriptional silencing (4) Involved with resistance to TGFβ signaling in AML (54) Deregulation of direct TGFβ targets p21 and p15—inhibiting cell cycle arrest through constitutive H3K9me3 (55, 56) Physical Interaction with proto—oncogenes (57–60) Creates a binding site for HP1 gene silencing, also allowing DNA methylation (61)
SETDB1/2	Methyltransferase	H3K9me3	Major role in preventing immune presentation through heterochromatin formation—allowing evasion of immune response (62–65) Functions through PAF 1 pathway regulating fundamental leukaemogenesis genes via Wnt Signaling (66)
KDM4	Demethylase	H3K9me3	Direct role to disrupt the cellular proliferation equilibrium, genomic instability and replication acceleration due to an increased accessibility to chromatin, risking the introduction of further mutations (67–69). Indirect disruption due to downstream targets of aberrant methylation for examples I3ra and JAK Stat pathway (69, 70)
PRC Complex	Reader Complex	H3K9me2/3	Involved in reading and regulation of epigenetic modification, depletion of members of the complex result in overall decrease in H3K9me3 and a subsequent relocation and change in HP1 stability (71)
UHRF1	Regulator	H3K9me 1/2/3	Regulates the interaction of H3K9 methylation modifications with DNA methylation
G9a/GLP	Methyltransferase	H3K9me2	Involved in transcriptional silencing, essential for embryonic development, and restriction of lineage during haematopoietic development
LSD1	Demethylase	H3K9me3	Known to demethylate H3K9me3 in an androgen receptor mediated manner, identified in androgen dependent prostate cancer affecting transcription. Also has known interactions with fundamental demethylases KDM4B/C (72–77)
PRDM Family	Methyltransferase	H3K9me2	Varying roles both directly and indirectly mainly against H3K9me2 through the G9a mediated pathway. PRDM9 functions during meiosis to identify and recruit methyltransferases during double strand break repair (78–81). PRDM14 and PRMD2 function in an indirect manner whilst PRDM8 has a methyltransferase activity identified (82, 83)

#### Writers of H3K9me3 Modifications

##### SUV39H1

SUV39H1 and SUV39H2 were the earliest methyltransferases identified. Of initial interest due to their respective homologs in model organisms, *D. melanogaster and S. pombe*, being associated with position effect variegation (PEV) which leads to the relocation of genes into regions of heterochromatin and subsequent transcriptional silencing. Sharing a 59% sequence identity, belonging to the Su (var) gene family and specifically methylating H3K9 (4, 84), these methyltransferases possess two evolutionarily conserved domains, the N-terminal chromo domain (CD) and the C-terminal suppressor of variegation, enhancer of zeste and trithorax (SET) domain. The methyltransferase catalytic activity of the SET domain was elucidated using an *in vitro* systematic approach, whereby truncated versions of *SUV39H1* were tested to observe which conserved regions were required to successfully methylate free histone. This approach also identified H3K9 as the transferases main substrate (4), other studies have shown that by genetically reducing the levels of SUV39H1 there is an observed decrease in global H3K9 methylation (85, 86). Opposed to this but still fundamental for activity the N terminus chromodomain facilitates recruitment of the transferases by binding trimethylated H3K9 and allows the establishment of broad heterochromatin domains due to the successive local spreading of H3K9me3 (25). Interestingly, the spreading mode of H3K9me3 due to SUV39H1 has been explored further, utilizing innovative *in vitro* synthetic chromatin which closely resembles natural chromatin demonstrating that SUV39H1 first binds its target H3K9 and then engages with the chromatin through a second zinc finger like-domain which acts to enhance its catalytic activity (87). Shirai et al. have also revealed that the CD of SUV39H1 is able to efficiently bind RNA in an H3K9me3 independent manner and that this contributes to efficient recruitment to chromatin (20, 88).

Alongside its role in normal stability SUV39H1 was also the first histone lysine methyltransferase to have its role in AML pathology characterized. One major pathway implicated in the progression of AML and a significant pathway in the regulatory function of SUV39H1 is the acquired resistance to Transforming Growth Factor beta (TGFβ). TGFβ is a signaling pathway frequently deregulated in haematopoietic malignancies with anti-proliferative and differentiation signaling demonstrated to directly arrest growth and inhibit colony formation in the leukaemic stem cell population. Thought to be due to a reduction in receptors on the cell surface as opposed to a mutation, resistance to TGFβ signaling is commonly observed in AML phenotypes (54, 89, 90). MDS1 and EVI1 Complex Locus Protein (MECOM), a potent proto-oncogene known to be involved in stem cell self-renewal and leukaemogenesis physically interacts with the SUV39H1 histone methyltransferase and has been implicated in disease progression of AML in around 5% of cases and its expression negatively correlates with survival in AML. Despite EVI1 not being essential for the methylation functions of the SUV39H1 molecules, this co-localization is necessary for transcriptional repression of the TGFβ pathway associated with the EVI1 AML phenotype. Similarly, the activity of EVI1 requires co-activation of the HOXA9 gene to drive leukaemogenesis, a gene which has been investigated with respect to the demethylase enzyme family and target genes (57–60). Targets of the TGFβ pathways are frequently deregulated in AML. For example, p15 and p21, major tumor suppressors and targets of TGFβ are silenced in AML by pathways involving SUV39H1, giving the leukaemic cells an advantage to proliferate. The gene repression of p15 locus observed in AML is reliant upon several differential epigenetic marks. Aberrant di-methylation of H3K9 on the promoter region of p15 in AML is dependent upon DNA methylation at the promoter region and a specific acetylation pattern on H3K9. A constitutive level of H3K9me3 is required to repress p15 and establish an AML phenotype.

Similarly, an aberrant epigenetic regulation landscape is observed in AML when the expression of p21 is altered, through the action of SUV39H1. Although not carried out on haematopoietic cell lines, Cherrier et al. studied the interaction of SUV39H1 with CTIP2 also known as B-cell lymphoma/leukemia 11B (BCL11B). By increasing H3K9me3 so reducing p21 gene expression, this SUV39H1/CTIP2 complex allows proliferation of leukaemic cells and their failure to undergo apoptosis. BCL11B has been shown to be aberrantly expressed in AML (91, 92). By inhibiting the SUV39H1 induced H3K9me3, p21 facilitated cell cycle arrest can be initiated. p15 and p21 post-translational histone modifications can be restored to normal using an SUV39H1 inhibitor such as chaetocin, which will be discussed in more detail later in this review (55, 56, 61, 93). SUV39H1 dependent methylation of H3K9 creates an enhanced binding site for the HP1 protein, a known gene silencing and enhanced heterochromatin associated protein. This results in an inhibition of gene transcription (61, 94). By creating a complex together, they have been shown to interact with DNA methyltransferases, specifically DMNT3. Originally identified in *D. melanogaster* (95) loss of specific DNA methylation genes elicit a dual role as H3K9 methylation was also reduced. By investigating the interactions of DNMT and HMT with the HP1 further, a larger network of epigenetic regulation begins to unfold. For example in AML a large crosstalk between epigenetic regulators develops, methylation of H3K9 creates a binding site for HP1 which allows the recruitment of DNMT catalyzing DNA methylation recruiting methyl-CpG-binding proteins which create a location for Histone Deacetylases (HDACs), priming H3K9 sites for methylation (96).

##### SETDB1/2

SET domain, bifurcated 1 (SETDB1) and SET domain, bifurcated 2 (SETDB2) represent a second family of methyltransferases (97, 98) that specifically target H3K9. They were named after the observation of an intervening 347 amino acid sequence that splits their SET domains (99). These proteins unlike SUV39H family lack a CD, instead possessing methyl-CpG binding domains with SETDB1 additionally possessing a triple Tudor domain (3TD), the function of which has recently been elucidated by crystallographic studies. Revealed to bind a novel bivalent chromatin state defined by H3K9me1/2 and K14ac it subsequently mediates the trimethylation of H3K9 (62). Overexpressed in several cancer phenotypes SETDB1 is necessary for the survival of over 70% of tested AML cell lines. By knocking down SETDB1, AML cells will apoptose via the removal of H3K9me3 on transposable elements, their subsequent expression, and the induction of the interferon immune response identifying it as a fundamental leukaemic cell survival protein (63). One of the major contributing factors of histone methyltransferases in the development of a leukaemic phenotype is their interaction to allow the evasion of the immune system response. Through increasing H3K9me3 at select retrotransposon sites SETDB1 increases the formation of heterochromatin preventing immune presentation allowing leukaemic cells to evade the immune response (63–65). A recently published paper elucidated a role for SETDB1 in the regulation of H3K9me3 through the PAF1 gene known to be associated with histone methylation in Mixed Lineage Leukemia (MLL). By physically interacting with MLL, PAF1 plays a role in regulating fundamental leukaemogenesis genes, for example *MEIS1* and the previously described *HOXA9*. Through critically regulating the Wnt/β-catenin pathway samples with high SETDB1 levels showed a significant reduction in both *MEIS1* and *HOXA9* by regulating and increasing the silencing associated with the H3K9me3 mark on these specific oncogenic gene promotors (66). Loss of SETDB1 has been identified to cause the differentiation of cells in the embryonic lineage (21).

#### Erasers of H3K9me3 Modifications: KDM4 Family of Histone Demethylases

At the same time as the methyltransferase enzymes were being investigated, three independent research groups provided insight into the α-ketoglutarate (α-KG), Fe^2+^, and O_2_ dependent lysine-specific demethylase 4 (KDM4) JmjC-domain containing demethylase family of proteins, whose catalytic activity reverses the H3K9me3 mark (100–102). Although their function was first characterized by these landmark studies, the KDM4 family was initially described *in silico* showing the family to be composed of six members (KDM4A-F), four functional members with KDM4E/F hypothesized as pseudo-genes. Each member ubiquitously possesses the JmjC and JmjN domains with KDM4A-C having an additional two Tudor domains (103). KDM4 proteins utilize a distinct hydroxylation chemistry to demethylate H3K9me3 unlike previously identified demethylases such as KDM1A which use a FAD-dependent amine reaction, making their targets mono/di methylated and preventing them from removing the trimethyl marks.

The structural disparity between the KDM4 members has functional consequences. For example, whilst the KDM4A-C proteins primarily function to demethylate trimethylated H3K9, KDM4D also efficiently demethylates H3K9me2 (104). To discern the essential amino acids and domains of the KDM4 family that are required for their catalytic activity, numerous site-directed mutagenic (105–107) and protein-truncation (102) strategies have been employed. These primarily concern KDM4A and have shown that both the JmjC and JmjN domains are required for demethylation, whereas the plant homeodomain and Tudor domains are not obviously necessary for catalytic activity and are thought to primarily facilitate recruitment and adhesion to specific chromatin loci to remove H3K9me3. For example, crystallographic studies have offered insight concerning the molecular mechanisms of KDM4 proteins and revealed key amino acid residues that determine substrate specificity in the catalytic site of KDM4A which interacts with H3K9me3 (106, 108, 109). Recent work has also shown that the tandem Tudor domain of KDM4C can also bind to H3K4me3 and that this interaction acts to enhance the demethylation of H3K9me3 on the same histone tail, owing to an increased affinity for the mark (110). Another important characteristic recently uncovered is the ability of KDM4A to function as an oxygen detector, restricting H3K9me3 demethylation in hypoxic conditions (111, 112). An unexplored feature of this family is their capability to form homodimers and heterodimers between themselves and the impact this has upon their regulation of H3K9me3. Initial work using a reporter construct suggests that heterodimer formation can synergise or neutralize the transcriptional impact of these proteins at specific gene targets depending on the dimer composition, for example KDM4C and KDM4D heterodimers can synergise to promote higher expression of target genes (104).

Importantly, the KDM4 subfamily is commonly overexpressed in human cancers disrupting the equilibrium of cellular proliferation (67). Given the role of the KDM4 family in demethylating trimethylated H3K9 and the distinct deregulation known for this mark in AML, it is necessary to fully investigate their mechanisms in leukaemogenesis (68). KDM4A has been associated with genomic instability resulting in accelerated replication due to the increase in chromatin accessibility and hence the possibility for the introduction of DNA replication errors to further progress disease (69). It has been investigated and implicated in the disease progression of several cancers via alternative pathways (39, 113–115). Upon knock-out of the KDM4 family (KDM4A-C) a decreased proliferation and colony forming potential of MLL-AF9 leukaemic cells was observed with prolonged survival of murine models (70). ChIP sequencing analysis upon knocking down the KDM4 family members identified thousands of regions which were indicative of KDM4A binding sides, of which 77% were within 1 kb of transcriptional start sites (TSS). A number of these sites show largely increased H3K9 methylation upon deletion of KDM4A-C and localization to sites which are positive for H3K4, demonstrating the crosstalk between modifications. A large amount of transcriptional changes were subsequently observed with 55 genes being up-regulated and 94 genes being down-regulated, with approximately half being associated with binding sides of the KDM4 family (70, 116). The molecular mechanisms elucidated to date in AML suggest KDM4A has roles both directly, dependent on its demethylase activity, and indirectly, through downstream targets of its methylation changes, in oncogenesis. In MLL-AF9 driven leukemia KDM4A has been demonstrated to be involved in the proliferation of leukaemic cells via the aberrant demethylation of the TSS region of *l3ra (cd123*), the α subunit of the heterodimeric IL-3 receptor that, together with the β subunit (IL3 receptor β), forms a functional high-affinity receptor. *S*ubsequent upregulation of downstream targets and the activation of the Janus Kinase/Signal Transducer and Activator of Transcription (JAK/STAT) pathway drive proliferation (69, 70, 117).

#### Readers of H3K9me3 Modifications

Writers and erasers of histone modifications functions are not exclusive, alongside their dynamic control of the mark they also have a unique and highly controlled feedback system by which they recognize and read the marks to allow for changes in the system to be identified, altered and maintained. H3K9me3 can be read by a number of domains, the PhD and Tudor domains function in the demethylases and the chromo domain which has previously been described in the SUV family of methyltransferases. Others will also contain ankyrin repeats or MBT domains allow them to read and regulate their own function (118). Whilst the core regulatory apparatus governing H3K9me3 levels is composed of methyltransferases and demethylases, another layer of control exists to permit greater flexibility in H3K9me3 regulation. For example, many proteins which belong to the Polycomb Repressive Complexes (PRC) have been implicated in regulating H3K9me3. Depletion of SUZ12, an essential member of the PRC2 complex results in an EZH2-independent simultaneous decrease in overall H3K27me3 and H3K9me3 levels and results in relocation of the HP1α isoform. Whilst the exact mechanism is still elusive, given their localization and demonstrated *in vitro* (cell line) interactions, SUZ12 is thought to stimulate SUV39H1 activity or be involved with either HP1α recruitment or stability (71). In addition, CBX7, a member of the PRC1 complex has also been shown to play a role in the stability of H3K9me3 at specific loci in a molecular mechanism requiring SUV39H2 recruitment (119).

### Complex Regulatory Network

Despite the physical accessibility of the histone modification site being essential, nucleosome occupancy has been identified as a key player in influencing the histone modifications and the complex network of crosstalk they employ to control gene expression and maintain cell identity (51). Transcriptional activation requires both the addition of post-translational marks which promote activation and the removal of post-translational marks which induce repression. A number of enzymes work together to achieve this, each requiring the other to gain the desired effect (120). For example, in AML the protein arginine methyltransferases PRMT4/5 are commonly overexpressed and contribute to the blockage in myeloid differentiation. PRMT1 is a necessary member of the MLL transcriptional complex (116), whilst PRMT6 inhibits the action of MLL to methylate H3K4. Similarly, acetylation of histone complexes (histone acetylates (HATs) have known interactions with the methylation of H3K9 (121). TIP60, which is reduced in AML, has a tumor suppressor role through its recognition of H3K9me3 through the activation of ATM kinase signaling and DNA damage checkpoint activation; it also interacts with p53 protein to initiate apoptosis of damaged cells (122). Similarly competing evidence has been published on the role of H3K9me3 in the establishment and maintenance of specific DNA methylation patterns (123). Originally identified in plant species trimethylation of H3K9 has been shown to directly affect cellular DNA methylation. DNA methylation is governed by two main families of enzymes, Methyl CpG-binding (MBD) family and BR-C, ttk and bab (BTB)/Pox virus and Zinc finger (POZ) family proteins, however these families do not exclusively interact with DNA methylation but also through histone modifications such as histone methylation and acetylation (123). One mechanism of this interaction involves HP1 binding leading to the recruitment of DNA methyltransferases adding a new layer of epigenetic control to the story. HP1 specifically binds during the cell cycle interacting specifically with H3K9me3 recruiting the DNA methyltransferases. During mitosis the binding with HP1 is displaced by the phosphorylation of H3 interrupting the mechanism of DNA methylation (REF 105). A more recent discovery of the ubiquitin-like, PHD, and RING finger containing 1 protein (UHRF1), found a more significant connection between histone methylation and DNA methylation with each reliant on the other for normal function (124). Similarly to the KDM4A family UHRF1 has a Tudor and Plant Homeodomain (PhD) with the former being responsible for binding the histone methyl marks. However, unlike the histone demethylase family UHRF1 has a secondary binding site specific for hypomethylated DNA foci (125). UHRF1 association with methylated H3K9me3 is required for DNA methylation maintenance binding hemimethylated DNA during S phase at the replication fork and recruiting DNA methyltransferases (124). Mutants of UHRF1 which are selectively unable to bind H3K9 methylation or hemi-mCpG, but not both, show a partial defect in heterochromatin, and an ability to bind DNMT1 with a partial rescue of DNA methylation showing that these functions are not mutually exclusive rather that sequence and modification state of DNA can play a part in histone modifications and vice versa (123, 125). These marks must all work together to hold the cell in a homeostatic position; evidence has shown that in AML these mechanisms lose their control and deregulation leads to the phenotype of disease (118).

H3K9me3 is the most prominent and fundamental component to the epigenomic outlook of AML however it is not the only form of histone methylation shown to have influence on the epigenomic and genomic landscapes in AML and other cancer phenotypes. H3K9me3 methyltransferases and demethylases have been shown to form a larger complex and both individually and interdependently influence the epigenetic landscape SETDB1 and SUV39H1 both discussed form a complex with di- and mono-methylase proteins G9A and GLP. In this manner all three forms of H3K9 methylation ultimately rely on the others for maintenance (126). Dimethylation of H3K9 has a major role in transcriptional control and is shown to be erased from tumor suppressors following successful treatment. Specifically in AML large sections of H3K9me2 are hypothesized to promote chromosome instability and mutagenesis changing transcription and silencing tumor suppressors (127). G9a/GLP heteromeric complex is essential for mammalian H3K9 methylation, specifically mono- and di-methylation. With aberrant gene expression following genetic knockdown of both or either of these genes they are hypothesized to be involved in transcriptional silencing (128). The complex is essential for embryonic cell development, lineage commitment, and restriction of reprogramming with spreading H3K9me2 observed during haematopoietic cell lineage development (129, 130). Inhibition of G9a/GLP reduces differentiation, promotes stem cell characteristics delaying disease and reducing LSC frequency via the leukaemic transcription factor HOXA9, implicating a crossover role with the output of H3K9me3 on HOX genes (131, 132).

A related phenomenon within the histone methylation story involves LSD1. LSD1 is a demethylase commonly associated with the demethylation of the activating mark of H3K4 methylation, despite this an alternative role has been postulated for its role in the demethylation of H3K9me3 (72). In the presence of androgen specific receptors LSD1's function alters to selectively control H3K9 methylation resulting in gene repression of androgen specific markers (73, 74).

Originally identified in normal and cancerous prostate cells, LSD1 interacts with androgen nuclear receptor and stimulates androgen receptor dependent transcription via its interaction with a number of factors which together, both as direct effectors or in a combinational manner contribute to the altered transcriptional outlook of disease (75).

Therefore, depending on its associated co-factor LSD1 can either be involved in activation accessibility to chromatin or as a repressive. Similarly this functions in a reciprocal manner, by inhibiting the function of LSD1 in androgen receptor dependent phenotype androgen receptor binding is altered (76). The direct mechanism of this ability to influence the substrate specificity of LSD1 is largely unknown however roles have been postulated for other post translational modifications such as phosphorylation and protein kinases. Similarly a dual interaction has been identified of LSD1 with KDM4C in an androgen receptor mediated system (77).

At a higher level than direct methylation of H3K9, many of the histone modification enzymes are under a form of regulation by the PRDM family of proteins. Themselves with varying known roles in maintaining H3K4 and H3K36 methylation they are thought to recruit intrinsic histone methyltransferases and demethylases to H3K9 influencing methylation status (82). Containing a similar domain to the SET domain conserved within the demethylase family known as PR domain, they interact with H3K9 methylation with varying effects. A few members have been postulated to have roles either directly or indirectly (133). PRDM9 with its main role in the identification and tethering of recombination hotspots during meiosis, binds and recruits other proteins into a multi-protein complex interacting with G9A, previously described as a H3K9me2 methyltransferase and CDYL a reader of the di methyl mark (78–82). PRDM2 is one of few members which have an identified histone methyltransferase activity and is known to work in an estrogen activation dependent manner mediating promotor repression via H3K9 methylation (82). Again PRDM14 functions in a pathway through the G9a protein in early stem cell and embryonic development and has a role not only in H3K9me2 maintenance but also H3K27me3 levels hypothesized to have a role in AML (82). In contrast to this indirect method PRDM8 is thought to have an intrinsic methyltransferase activity in mouse tissue but the function of the human homolog has not been widely explored (82, 83).

Thus, elucidating the regulatory mechanisms contributing to the multifaceted role of H3K9me3 in hematological malignancies will provide insights in aid of novel anti-leukaemic therapy development.

### Therapeutic Relevance of H3K9 Methylation and Its Regulators in AML

#### H3K9 Methylation Status Can Determine Survival in AML

In AML, specific histone methylome patterns owing to the deregulation of histone methyltransferases and demethylases bring with them a phenotype of disease, this could prove to be key in assessing patient's outcome and treatment regime (69, 134). Recent work has been carried out with the aim of improving the current prognostic diagnosis of AML via the incorporation of both epigenetic factors and genetic markers (135). In terms of histone methylation, it has been determined that global epigenome changes can refine the disease classification system in a large cohort of AML patients. Over 2000 genomic loci, around 453 promoters were found to have differential expression owing to changes in H3K9me3. It was then further investigated if these changes could distinguish the cell's origin and if a signature of histone modifications could effectively predict the outcome of the disease. By comparing AML to Acute Lymphoblastic Leukemia (ALL) and normal white blood cell (WBC) populations it was found that independent of karyotype, age or mutation status, promoter H3K9me3 levels correlated with the outcome of the disease in >70% of cases (13).

#### H3K9 Methylation Regulators as Potential Therapeutic Targets in AML

A better understanding of the regulatory pathways which govern this dynamic mark has continuously proven to be key in the precise selection of fundamental functional targets. The increasing importance of post translational regulatory proteins highlight them as potential therapeutically targets. Lysine-methylation and other modifications are complex networks which will take significant time to completely reveal its mechanisms (136). However, a number of studies and groups both industrial and academic are currently focused on collaborating to develop synthetic, organic and peptidomimetric chemistry to produce a wide variety of inhibitors that are capable of targeting epigenetic regulating enzymes. While great progress is being made, selectivity remains a significant challenge.

#### Inhibitors of Writers of H3K9 Methylation as Novel Therapeutic Agents^1^

**Figure 2:**
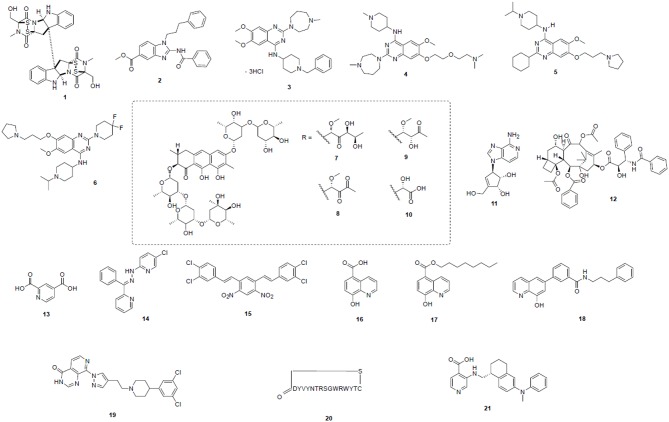
Potent epigenetic inhibitors targeting H3K9 modifying enzymes—including SUV39H1/2 (**1**), G9a (**2**–**6**), SETDB1 (**7**–**12**), and KDMs (**13**–**21**). Compound structures are numbered according to Table 2 and throughout the text.

Originally, Chaetocin **1** (Figure 2; Table 2) was reported as the first specific inhibitor for SUV39H1/H2, with an IC_50_ of 0.8 uM (61, 138). However, follow-up studies showed that this inhibition was non-specific and time-dependent, therefore, Chaetocin was unsuitable to act as a selective chemical probe for histone lysine methyl transferases (139, 140, 143). Most current drug developments involve targeting G9a including competition with the S-adenosylmethionine (SAM) cofactor for example inhibitor BRD4770 **2** (144). It is postulated that G9a/GLP, SUV39H1, and SETDB1 coexist in the same multi-protein complex together with other post-translational modifying proteins. Having an interdependency on one another knockdown or inhibition of SUV39H1 has the ability to not only also destabilize the G9a,GLP, and SETDB1 proteins but also affect the downstream targets of these proteins independently with a causal effect in histone methylase levels and activity (126). High throughput screening of over 125,000 compounds identified BIX01294 **3**, one of only seven compounds which showed any efficacy via inhibition of G9a/GLP against H3K9me2 (145). It was selected for further analysis due to its alternative method of binding in comparison with non-specific generic analogs which inhibit via cofactor S-adenosyl-methionine (SAM), such as methylthio-adenosine (MTA), sinefungin, and S-adenosyl-homocysteine (SAH) binding (141). However, it was found to have a high degree of toxicity against human cells restraining its potential (141). Using the 2,4-diamino-6,7-dimethoxyquinazoline template, Kim et al. developed UNC0632 **4**, a selective G9a and GLP inhibitor that was less toxic but also less potent than BIX01294 because of poor cell permeability (146).

**Table 2:** Overview of ^a^Assay as described by Eskeland et al. (137) ^b^AlphaLISA assay detecting H3K9 methylation.

**Entry**	**Name**	**Therapeutic target**	**IC50[ref](μM)**	**EC50[ref](μM)**	**Cell-line**
**1**	Chaetocin	SUV39H1/2	0.8 (117)^a^, 0.11 (118)^b^		
		G9a	2.5 (71)^b^, 7.2 (134)^b^		
**2**	BRD4770	G9a	6.3 (126)^h^		
**3**	BIX01294	G9a	0.018	0.0166 (135)^c^	NCl-H1437
		GLP	0.034	0.0147 (135)c	NCl-H1395^e^
**4**	UNC0321	G9a	<0.015	0.042 – 0.101 (138)^c^	A549^d^
		GLP		0.28 ± 0.11 (139)^f^	
**5**	UN0638	G9a	<0.015		e
		GLP	0.019 ± 1		
**6**	UNC0642	G9a	<0.0025	8.7 ± 0.37 (139)^e^	A549
		GLP			
**7**	MTM	SETDB1		0.0166 (135)^c^	NCl-H1437
				0.0147 (135)^c^	NCl-H1395^d^
**8**	MTM SDK	SETDB1		0.107–0.311(136)	e
				0.024–0.089 (138)^c^	
**9**	MTM SK	SETDB1		0.042–0.101 (138)^c^	e
				0.28 ± 0.11 (139)^f^	A549
**10**	MTM SD	SETDB1		8.7 ± 0.37 (139)^e^	A549
**11**	DZNep	SETDB1		~ 10 (122)^c^	H1299
					A549
					H460
**12**	Paclitaxel	SETDB1		0.0025–0.0075 (140)	g
**13**	PDCA	KDM4A KDM4E	0.7 (132)^i^ 1.4 (132)^i^		
		KDM4A	0.445 ± 0.03 (72)^j^		
**14**	JIB-04	KDM4B	0.435 ± 0.07 (72)^j^		
		KDM4C	1.1 ± 0.2 (72)^j^		
		KDM4E	0.34 ± 0.05 (72)^j^		
**15**	NSC636819	KDM4A	6.4 (73)^i^	16.5 (73)^c^	LNCaP
		KDM4B	9.3 (73)^i^		
		KDM4A	2.0 (77)^i^	86 (82)^k^	HeLa
**16**	IOX1	KDM4C	0.6 (82)^l^		
		KDM4D	0.2 (82)^l^		
		KDM4E	0.3 (82)^l^		
**17**	Octyl – 8 –Hydroquinoline- 5 - Carboxylate	KDM4C	3.9 (133)l	3.8 (133)^c^	HeLa
		KDM4E	45.0 (133)^l^		
**18**	B3	KDM4B	~ 0.01 (78)^m^	0.04 (78)^c^	PC3
**19**	8-(1H pyrazol−3- yl)pyrido[3,4d]pyrimidin– 4(3H)-one	KDM4A	0.08 ± 0.042 (79)^l^		
		KDM4B	0.017 ± 0.002 (79)^l^		
		KDM4A	42 (80)^m^		
		KDM4B	33 (80)^m^		
**20**	CP2	KDM4C	39 (80)^m^		
		KDM4D	6,270 (80)^m^		
		KDM4E	9,200 (80)^m^		
**21**	QC6352	KDM4C	0.035 ± 8	0.0035 ± 1	

c *MTT assay*.

d *WST1 assay: MSC-4H and T-4H (E) or MSC-5H and T-5H cell lines*.

e *DU145, 22RW1, PC3, LNCaP, A549*.

f *Resazurin assay. ^g^HeLa, A549, U373, MCF-7, HT-29, OVG-1, PC-Sh, PC-Zr*.

h *DELFIA assay (141)*.

i *FDH coupled assay*.

j *ELISA assay*.

k *Assay as described by King et al. (142)*.

l *AlphaScreen*.

m *Histone demethylase assay*.

To improve the cell permeability they utilized a previously established structure activity relationship of the quinazoline scaffold to design a new generation of inhibitors with better cell permeability while maintaining the same potency. Among these compounds UNC0638 **5** had the best potency and physiochemical potency and subsequently UNC0642 **6** a selective inhibitors of EHMT2 (G9a) inducing a selective H3K9me2 decrease (146).

Although the importance of SETDB1 as a potential therapeutic target was recently demonstrated, selective inhibitors for SETDB1 have not been extensively described (147). A number of potential nonspecific inhibitors including mithramycin (MTM, **7**) and its analogs eg. MTM SDK **8**, MTM SK **9**, and MTM SD **10**, together with the cyclopentenyl analog of 3-deazaadenosine, DZNep **11** have been postulated. The latter non-specifically inhibits histone methylases (148), and a decreased methylation of H3K9 was observed in the nucleus of DZNep-treated cells. Transcription of SETDB1 can be regulated by DZNep and its expression was decreased in lung cancer (149). Similarly Paclitaxel **12** is a, widely used anti-cancer drug and downregulation of SETDB1 at the transcriptional level by **12** resulted in the death of human lung cancer cells (150). However, little is known about the effectiveness of these drugs against hematological malignancies.

#### Inhibitors of Erasers of H3K9 Methylation as Novel Therapeutic Agents

As previously described lysine specific demethylation in humans is catalyzed by two subfamilies of demethylases, namely, 2-oxoglutarate (2OG)—and flavin-dependent lysine demethylases. Their active sites consist of the JmjC- and amine oxidase-like (AOL) domains, respectively. Structural similarities and the conservation of active sites within the KDM families, makes design of specific inhibitors challenging. The concepts of KDM inhibition have been well-covered by Thinnes, Kaniskan and Lohse et al., who have described a wide variety of JmjC inhibitors (114, 136, 151). Specific inhibitors for KDM4 have been described in detail (67, 152). In AML with a major focus on H3K9me3 and specifically the KDM4A-D subfamily which catalyze its demethylation inhibitors of this specific family will be discussed here in more detail.

The KDM subfamily containing the JmjC domain has a Fe (II) containing catalytic site (153). Unlike the AOL domain, the JmjC domain is able to demethylate trimethyl lysine. The catalytic cycle requires the co-factors molecular oxygen and 2OG (154). Most inhibitors are small molecules and will, either act as 2OG mimics competing with 2OG, or act by chelating Fe^2+^ in the active site. Early promising metal binding inhibitors included pyridine-2, 4-dicarboxylic acid (PDCA, **13**), capable of selective inhibition of KDM4A and KDM4E (IC_50_ = 0.7 and 1.4 μM, respectively) over the other JmjC containing KDMs (155). In the search for potential inhibitors for 2OG-oxygenases, Wang et al. uncovered JIB-04 **14**, a hydrazone derivative. Although **14** was a potent inhibitor, it was not selective and a range of KDM family members were inhibited including KDM4A (IC_50_: 0.445 ± 0.03 μM) and KDM4C (IC_50_:1.1 ± 0.2 μM) (156). NSC636819 **15**, was uncovered via a structure guided compound screen with available docking and domain knowledge. Although it was capable of inhibiting KDM4's with an association for KDM4A (IC_50_ = 6.4 μM) and KDM4B (IC_50_ = 9.3 μM) it was not as potent as other inhibitors published at the time (namely JIB-04) (157) and in a prostate cancer model has a reduced inhibitory concentration of 16.5 μM showing a reduced cell permeability and specificity. **15** functions as a competitive inhibitor and despite have a relatively low efficacy against KDM4 family members may provide an alternative framework base for drug development. Similar to **14, 15** is also capable of inhibition of Prolyl-hydroxylase and HIF1α in the micromolar range (158). A clear disadvantage in inhibitor **15** is the presence of nitro aromatic moieties, which are potentially carcinogenic and therefore undesirable (159, 160). Interestingly downstream gene targets of prostate cancer cell lines treated with 11 showed a number of pathways alongside 27% androgen response gene targets identifying a possible crossover function of LSD1 and KDM4A/B.

High throughput screening of potential inhibitors for 2OG-oxygenases resulted in uncovering 8-hydroxyquinoline-5-carboxylic acid (IOX1, **16**), with an IC_50_ of 0.2 μM against KDM4A specifically (142). in addition to an EC_50_ value of in HeLa cells of 86 μM. Clearly, the polar character of IOX1 caused by the carboxylic acid moiety, hydroxyl, and amino groups hinder membrane permeability, explaining the >400-fold decrease in potency biochemical assays as compared to that in cells (161). To improve the membrane permeability, Schiller et al. (161) synthesized ester derivatives of **16** with varying lengths of alkyl groups. Evaluation of activity against KDM4A as a representative histone demethylase by immunofluorescence identified by transiently overexpressing KDM4A in a HeLa cell line model and then subsequently treating with each compound individually, H3K9me3 levels following treatment significantly increased on comparison with both control and overexpression of a mutant KDM4A protein, as a negative. They found that an n-octyl ester derivative, octyl 8-hydroxyquinoline-5-carboxylate **17** was superior to its parent compound likely due to increased membrane permeability (162). A more extended ALPHA screen assay was then used to compare the selectivity of **17** against other 2OG oxygenase families in comparison to their KDM4 function. The results support the classification of **16** as a broad-spectrum 2OG-oxygenases inhibitor (161). Compound **17** restricted the observed inhibitory activity to the KDM4 subfamily. Specifically, KDM4C was inhibited most potently with an IC_50_ value of 0.6 μM (161). **17** was also the least cytotoxic compound and the most potent inhibitor of H3K9me3 demethylation with an IC_50_ of 3.8 μM (162).

Additionally, an 8-hydroxyquinoline containing compound was utilized to inhibit KDM4B. 3-(8-Hydroxyquinolin-6-yl)-N-(3-phenylpropyl) benzamide (B3, **18**) inhibited KDM4B with an IC_50_ of ca. 10 nM in enzymatic studies and also significantly inhibited the enzymatic activity of other KDM4 isoforms (163). Another very potent KDM4A/B inhibitor class was developed by Bavetsias et al. (139) and comprised the *N*-substituted4-(pyridin-2-yl) thiazole-2-amine derivatives which were optimized to give 8-(1H-pyrazol-3-yl)pyrido[3,4-d]pyrimidin-4(3H)-ones **19**. Having a 4- (3,5-dichlorophenyl) piperidine linker this compound was the most potent in the series, capable of inhibition of KDM4A with an IC_50_ value of 80 nM and KDM4B with an IC_50_ value of 17 nM (164). Kawamura et al. reported cyclic peptide inhibitors of KDM4A-C demethylase enzymes with selectivity over other KDMs, including closely related KDM4D/E isoforms. CP2 **20** was uncovered as the most promising cyclic peptide. **20** selectively inhibited KDM4A-C with IC_50_ values of 42, 33 and 39 nM, respectively (165).

The KDM4 family are deregulated in many cancer phenotypes, one which has been the focus of drug discovery in recent times is breast cancer. Breast cancer stem cells as they have been termed have been postulated to be responsible for metastasis and resistance to current therapies. This stem cell population is poorly characterized but thought to involve KDM4 family members for maintenance. Using a 3D tissue model and cell free assays Metzger et al. (166) showed that knockdown of KDM4A had effect in breast cancer tumors in mouse models through its control of cell proliferation (166). They went on to utilize a compound identified by Chen et al. (167) known as QC6532 to chemically inhibit the action of KDM4 family members. QC6532 **(21)** was developed through a complex strategy of compound modification. Through screening of an established database they selected an appropriate base structure with known efficacy by substituting the carboxylic acid position they selectively increased hydrophobic interactions and improved cellular permeability, alongside optimizing the position of tetrahydronaphthalene to increase potency (167). Intermediate compounds showed favorable interactions with the KDM4 family and through using crystal structural analysis the group designed compounds substituting on the active site aromatic ring. QC6352 showed cellular activity with a relatively reduced potency however perceived activity against KDM5 family remained significantly high to an extent that it may influence the phenotype observed (167). It has promising efficacy against breast cancer cell lines and 3D culture models and encouragingly is readily orally available in mouse models with low clearance and a high volume of distribution (166). Other inhibitors such as A366 have been identified however as their main target so far has only been identified to be KDM4 family action on H3K4 methylation they are outwith the scope of this review (168).

## Conclusion

H3K9me3 is a dynamic covalent post-translational histone modification which is known to be pathologically deregulated in a number of diseases including haematopoietic malignancies. It is tightly controlled by a number of enzymes which place, maintain and remove H3K9me3 such as the KDM4 family which are rapidly emerging as factors which demonstrate a strong regulatory influence on oncogenic gene transcription in AML. Whilst progress has been made in targeting and developing of inhibitors for the specific H3K9me3 methyltransferases or demethylases, selectivity remains an unmet challenge. The worrying statistics which accompany AML with a 5 year survival rate of <40%, highlight the necessity to continually drive to identify new mechanisms for the development of *de-novo* AML both causative and as consequences of driver mutations. Alongside this identifying and developing the ability to target these functions specifically. A comprehensive understanding of the oncogenic function of H3K9me3 within AML cells and the respective epigenetic regulators that fine-tune its abundance will permit the development of more efficient therapeutic strategies in the future.

## Author Contributions

LM, MM, RB, AH, and P-AP drafted the manuscript and prepared figures. RL, HJ, and XH critically analyzed and revised the manuscript to its final form. All authors read and approved the final manuscript.

### Conflict of Interest Statement

The authors declare that the research was conducted in the absence of any commercial or financial relationships that could be construed as a potential conflict of interest.

## Abbreviations

AML: Acute myeloid leukemia
H3K9me3: Histone 3 lysine 9 trimethylation
HSC: Haematopoietic stem cells
LSC: Leukaemic stem cell
HP1: Heterochromatin protein 1
TSS: Transcriptional start site
HAT: Histone acetylates
PEV: Position effect variegation
CD: Chromo domain
SET: Suppressor of variegation, enhancer of zeste and trithorax
TGFβ: Transforming Growth Factor beta
MECOM: MDS1 and EVI1 Complex Locus Protein
BCL11B: B-cell lymphoma/leukemia 11B
HDAC: Histone Deacetylases
SETDB1: SET domain, bifurcated 1
SETDB2: SET domain, bifurcated 2
MLL: Mixed Lineage Leukemia
α-KG: α-ketoglutarate
KDM4: Lysine-specific demethylase 4
JAK/STAT: Janus Kinase/Signal Transducer and Activator of Transcription
PRC: Polycomb Repressive Complexes
ALL: Acute Lymphoblastic Leukemia
WBC: Normal white blood cell
SAM: S-adenosylmethionine
2OG: 2-oxoglutarate
AOL: JmjC- and amine oxidase-like.
